# Perfluorooctane sulfonate induces autophagy-associated apoptosis through oxidative stress and the activation of extracellular signal–regulated kinases in renal tubular cells

**DOI:** 10.1371/journal.pone.0245442

**Published:** 2021-01-20

**Authors:** Li-Li Wen, Yen-Ting Chen, Yuan-Chii Gladys Lee, Tsui-Ling Ko, Hsiu-Chu Chou, Shu-Hui Juan

**Affiliations:** 1 Graduate Institute of Medical Sciences, Taipei Medical University, Taipei, Taiwan; 2 Department of Clinical Laboratory, En Chu Kong Hospital, New Taipei City, Taiwan; 3 Department of Physiology, School of Medicine, College of Medicine, Taipei Medical University, Taipei, Taiwan; 4 Graduate Institute of Biomedical Informatics, College of Medical Science and Technology, Taipei Medical University, Taipei, Taiwan; 5 School of Medicine for International Students, College of Medicine, I-Shou University, Kaohsiung, Taiwan; 6 Department of Anatomy and Cell Biology, School of Medicine, College of Medicine, Taipei Medical University, Taipei, Taiwan; Sechenov First Medical University, RUSSIAN FEDERATION

## Abstract

Perfluorooctane sulfonate (PFOS) is among the most abundant organic pollutants and is widely distributed in the environment, wildlife, and humans. Its toxic effects and biological hazards are associated with its long elimination half-life in humans. However, how it affects renal tubular cells (RTCs) remains unclear. In this study, PFOS was observed to mediate the increase in reactive oxygen species (ROS) generation, followed by the activation of the extracellular-signal-regulated kinase 1/2 (ERK1/2) pathway, which induced autophagy in RTCs. Although PFOS treatment induced autophagy after 6 h, prolonged treatment (24 h) reduced the autophagic flux by increasing lysosomal membrane permeability (LMP), leading to increased p62 protein accumulation and subsequent apoptosis. The increase in LMP was visualized through increased green fluorescence with acridine orange staining, and this was attenuated by 3-methyladenine, an autophagy inhibitor. N-acetyl cysteine and an inhibitor of the mitogen-activated protein kinase kinases (U0126) attenuated autophagy and apoptosis. Taken together, these results indicate that ROS activation and ROS-mediated phosphorylated ERK1/2 activation are essential to activate autophagy, resulting in the apoptosis of PFOS-treated RTCs. Our findings provide insight into the mechanism of PFOS-mediated renal toxicity.

## Introduction

Perfluorooctane sulfonate (PFOS) is a persistent organic pollutant that is widely distributed in the environment, wildlife, and the human body. PFOS is a source of concern because of its wide environmental distribution and long elimination half-life in humans [1]. We previously investigated the effects of PFOS on renal tubular cells (RTCs) and found an increase in the transcription of tumor necrosis factor-α, monocyte chemoattractant protein 1, and intercellular adhesion molecule 1, which activate nuclear factor κ-light-chain enhancer of activated B cells both in vivo and in vitro [2]. In addition, PFOS inhibited the expression of antioxidative enzymes, including glutathione peroxidase 1, superoxide dismutase 1, and catalase in RTCs, making them susceptible to oxidative stress. This further leads to increased apoptosis through increasing the Bcl-xS-to-Bcl-xL ratio and caspase 3 activation in RTCs [2, 3].

PFOS increases reactive oxygen species (ROS) production in normal thymus and spleen cells, hepatoma HepG2 cells [4], and lung cancer A549 cells [5]; it impairs mitochondrial membrane potential, leading to type I programmed cell death [6]. In addition, PFOS induces autophagy in hepatocytes [7]. Autophagy is a cellular physiological process that recycles damaged cellular components based on oxidized macromolecules and aged organelles. Autophagy occurs continuously in cells. During starvation, cells can provide self-nutrition through lysosomal enzyme–induced degradation of macromolecules and damaged proteins [8]. However, autophagy can act as a double-edged sword and induce cellular apoptosis through type II programmed cell death. Autophagy is initiated by the C-terminal cleavage of light chain 3 (LC3)–associated microtubule protein (LC3B peptide) by autophagy-related gene 4, which is a type of cysteine protease. It yields LC3BI, which conjugates with phosphatidylethanolamine (PE) to produce LC3BII [9], which can conjugate with autophagy-related proteins 5, 7, and 12 to produce autophagosomes along with phospholipid bilayers. By contrast, LC3 interacts with p62/sequestome-1 (SQSTM1), which acts as a ubiquitin-binding protein to degrade damaged organelles and macromolecules [10]. Two upstream molecules regulate autophagy induction: adenosine monophosphate (AMP)–activated protein kinase (AMPK) and phosphorylation target of rapamycin (pmTOR). During nutrient deficiency, to increase the ratio of AMP to adenosine triphosphate, AMPK is phosphorylated to trigger autophagy [11], whereas pmTOR suppresses the activation of autophagy [12].

Oxidative stress can act as an intracellular alarm reflecting the lack of availability of nutrients outside a cell, thus activating autophagy to provide nutrients and energy through recycling aged organelles and oxidized macromolecules, which in turn reduces oxidative stress through negative feedback regulation [13]. In this study, we investigated the mechanisms underlying the cytotoxic effect of PFOS on RTCs associated with autophagy activation and lysosomal membrane instability. We also investigated the prevention of this effect using N-acetyl cysteine (NAC).

## Materials and methods

### Cell culture and reagents

Rat proximal RTCs (NRK-52E) were purchased from the Bioresource Collection and Research Center (Hsinchu, Taiwan) and cultured in Dulbecco’s modified Eagle’s medium (DMEM) supplemented with an antibiotic–antifungal solution and 5% fetal bovine serum (FBS; pH 7.2). Cells were grown to 85%–95% confluence before use in a humidified incubator at 37°C, and cells from passages 5–20 were used for further analyses. FBS, DMEM, and tissue culture reagents were obtained from Invitrogen (Carlsbad, CA, USA). NAC and PFOS were purchased from Sigma Chemical (St. Louis, MO, USA). 3-Methyladenine (3-MA) and hydroxychloroquine sulfate (CQ) were obtained from Merck Millipore (Darmstadt, Germany), and 3-(4,5-dimethyl thiazol-2-yl)-2,5-diphenyl tetrazolium bromide (MTT) was purchased from SERVA Electrophoresis GmbH (Berlin, Germany). Protein assay agents were purchased from Bio-Rad (Hercules, CA, USA). The concentrations of chemicals and duration of treatments for each assay were determined according to our previous work [14–16] or pilot studies.

### Flow cytometry

Cells grown in a 10-cm Petri dish were stored in 0.5% FBS DMEM medium for 24 h, followed by 24/48-h treatment with PFOS in 5% FBS DMEM with or without 2-h 3MA pretreatment. The resulting cells were harvested and centrifuged at 1500 ×*g* for 10 min and washed twice with ice-cold phosphate-buffered saline (PBS). Cells were fixed in 1 mL of alcohol (70%) and maintained at −20°C for at least l h. Next, 1 mL of PBS-based reaction mixture containing propidium iodide (5 μg/mL), RNase (50 μg/mL), and 0.1% Triton X-100 (Sigma) was added to cell samples. The samples were then incubated in the dark for 30 min prior to the analysis of the sub-G1 phase and cell-cycle progression through flow cytometry.

### MTT assay

Cell viability was assessed based on the activity of mitochondrial dehydrogenase to reduce MTT to formazan. In brief, cells were seeded in 24‐well plates at 1.5 × 10^4^ cells/well and treated with dimethyl sulfoxide (DMSO) or 100 μM PFOS at indicated time points with or without 1 mM 3MA pretreatment for 2 h. MTT was then added to each well, and incubation was continued for 30 min at 37°C. Formazan formed was dissolved with DMSO for 30 min on a shaker, and absorbance was then measured at 570 nm using a spectrophotometer.

### Western blot analysis

RTCs were seeded in 6- or 10-cm dishes after the indicated treatments. The resulting cells were harvested for total cell lysates with the addition of protease inhibitors according to the manufacturer’s instructions. The cell lysate was centrifuged at 16 000 ×g at 4°C for 20 min, and the supernatant containing total protein was collected. The concentration of total protein was quantified using Bio-Rad protein assay agents. Antibodies for Beclin 1 (SC-48381, Lot: F2917), Bax (SC-526, Lot: B1115), phosphorylated extracellular-signal-regulated kinases 1 and 2 (pERK1/2; SC-7383, Lot: B1519), poly(adenosine diphosphate-ribose) polymerase (PARP; SC-74469, Lot: E1208), and β-actin ([SC-47778, Lot: G0213], 1:500, Santa Cruz Biotechnology, Santa Cruz, CA, USA); microtubule-associated protein LC3B (GTX127375, Lot: 42592) and nucleoporin p62/SQSTM1 ([GTX107973, Lot: 41059], 1:500, GeneTex, Irvine, CA, USA); and caspase 3 ([9662S, Lot: 18], 1:1000, Cell Signaling, Danvers, MA, USA) were included in the assay. Cell lysates (80 μg) were electrophoresed on 10%–12% sodium dodecylsulfate–polyacrylamide gel and transblotted onto an Immobilon PVDF membrane (Merck Millipore). The blots were then incubated with the aforementioned primary antibodies overnight at 4°C and then incubated with appropriate horseradish peroxidase–conjugated secondary antibodies (1:5000, Sigma) for 1 h at room temperature, which were detected using the Immobilon Western Chemiluminescent HRP Substrate (Merck Millipore) according to the manufacturer’s instructions. The expected protein bands were detected using the UVP BioSpectrum 500 imaging system (Analytik Jena US LLC, Upland, CA, USA). The relative abundance of the target protein was measured using ImageJ software and normalized to the β-actin band intensity.

### Immunofluorescence staining for lysosomal, cytosolic, and mitochondrial reactive oxygen species in PFOS-treated RTCs

The cells grown overnight on glass coverslips in 6-well Petri dishes were pretreated with 1 mM 3MA or 10 mM NAC for 2 h with or without an additional 100 μM PFOS treatment for 24 h. The resulting cells were subjected to 2 μg/mL of acridine orange (AO; Invitrogen) for the lysosomal membrane permeability (LMP) assay, with 2.5 μg/mL of 2′,7′-dichlorofluorescein diacetate (DCFH-DA, Sigma) for cytosolic ROS detection or with 5 μg/mL of MitoSox dye (Thermo) for mitochondrial ROS detection at 37°C for 15 min, followed by three PBS washes. The resulting cells were fixed in 4% paraformaldehyde and incubated with blocking buffer for 1 h. The cells were then permeabilized with 0.2% Triton and 0.1% Tween 20 in blocking buffer (3% bovine serum albumin in PBS) for 2 h. The MitoSox-stained cells were also counterstained for nuclei with 4′,6-diamidino-2-phenylindole (DAPI) dye. The fluorescence was measured using a charge-coupled device camera (DP72, Olympus, Melville, NY, USA) attached to a microscope system (BX51, Olympus) at 100× magnification. Four coverslips in each experimental group were examined. The subsequent procedures are described elsewhere [17].

### Statistical analysis

Data from at least three experiments are presented as mean ± standard error of the mean (SEM). The means between two conditions were compared using an unpaired Student’s *t* test and among multiple groups using a one-way analysis of variance or Bonferroni post hoc test. *P* < 0.05 was considered significant.

## Results

### Association between autophagy induction through PFOS and apoptosis in RTCs

**Fig 1:**
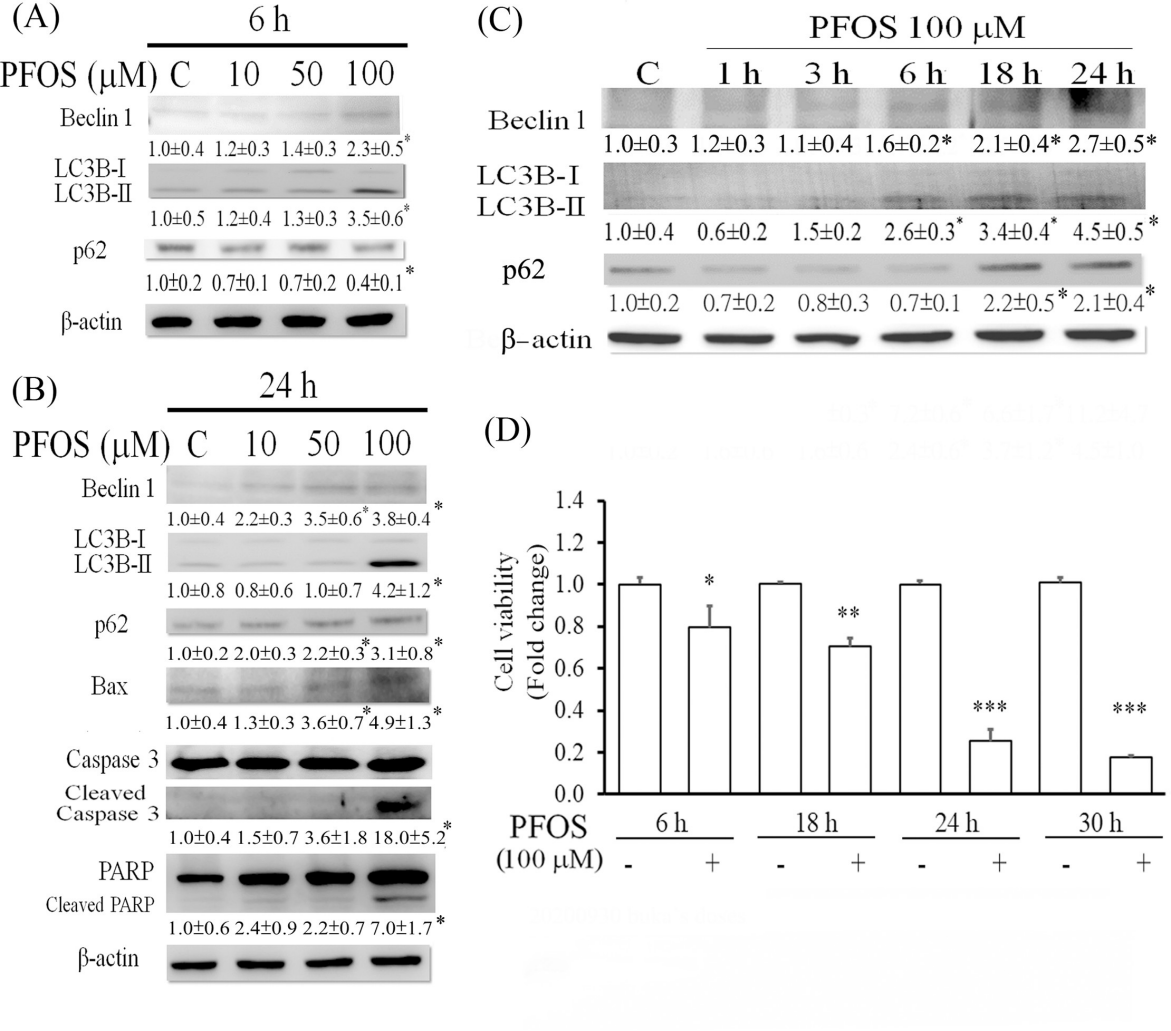
Concentration- and time-dependent effects of PFOS on the induction of autophagy and apoptosis in RTCs. Cells were treated with various concentrations (0–100 μM) of PFOS for 6 h (A) and 24 h (B) followed by Western blot analysis for proteins involved in autophagy induction and apoptosis. The intensity of each protein band was quantified through densitometry and normalized with an internal control of β-actin, and the value is presented as the mean ± standard error of mean (SEM) shown in each membrane blot (*P < 0.05 vs. control). (C) Western blot analysis was used to examine the time-dependent effect (0–24 h) of 100 μM PFOS on the protein expression of LC3BII and p62. (D) After 6–30-h PFOS treatment, RTC viability was measured using an MTT assay. Data shown are presented as the mean ± SEM of six independent experiments. *P < 0.05, **P < 0.01, and ***P < 0.005 vs the respective time-point control.

We previously demonstrated that PFOS acts as a toxic substance for RTCs [2, 3], resulting in apoptosis. To further characterize the underlying mechanism associated with apoptosis, Western blot analysis was performed to examine the concentration- and time-dependent effects of PFOS on autophagy and apoptosis. An increasing concentration (10–100 μM) of PFOS activated autophagy by increasing Beclin 1 and LC3BII levels and reducing the level of p62 protein, an autophagy substrate, after 6 h of treatment (Fig 1A). However, a prolonged 24-h PFOS treatment significantly increased p62 and LC3BII levels, accompanied by increased levels of apoptotic markers (Fig 1B), including cleaved caspase 3, PARP, and Bax. These results indicate that blocked autophagic flux and induced apoptotic cell death occurred in PFOS-treated RTCs after 24 h of treatment. This finding was validated by determining the time-dependent effect of 100 μM PFOS on autophagy (Fig 1C). PFOS initiated autophagy by increasing LC3BII and Beclin 1 levels after 6 h of treatment but significantly attenuated the autophagic flux by increasing p62 and LC3BII accumulation after 18 and 24 h of treatment (Fig 1C). Furthermore, an MTT assay revealed significantly decreased viability of cells treated with 100 μM PFOS at 6 h and 18 h, followed by a significantly greater extent of increase in cell death after 24 h and 30 h of treatment (Fig 1D).

### Autophagy activation followed by a decreased autophagic flux and apoptosis in perfluorooctane sulfonate–treated RTCs

**Fig 2:**
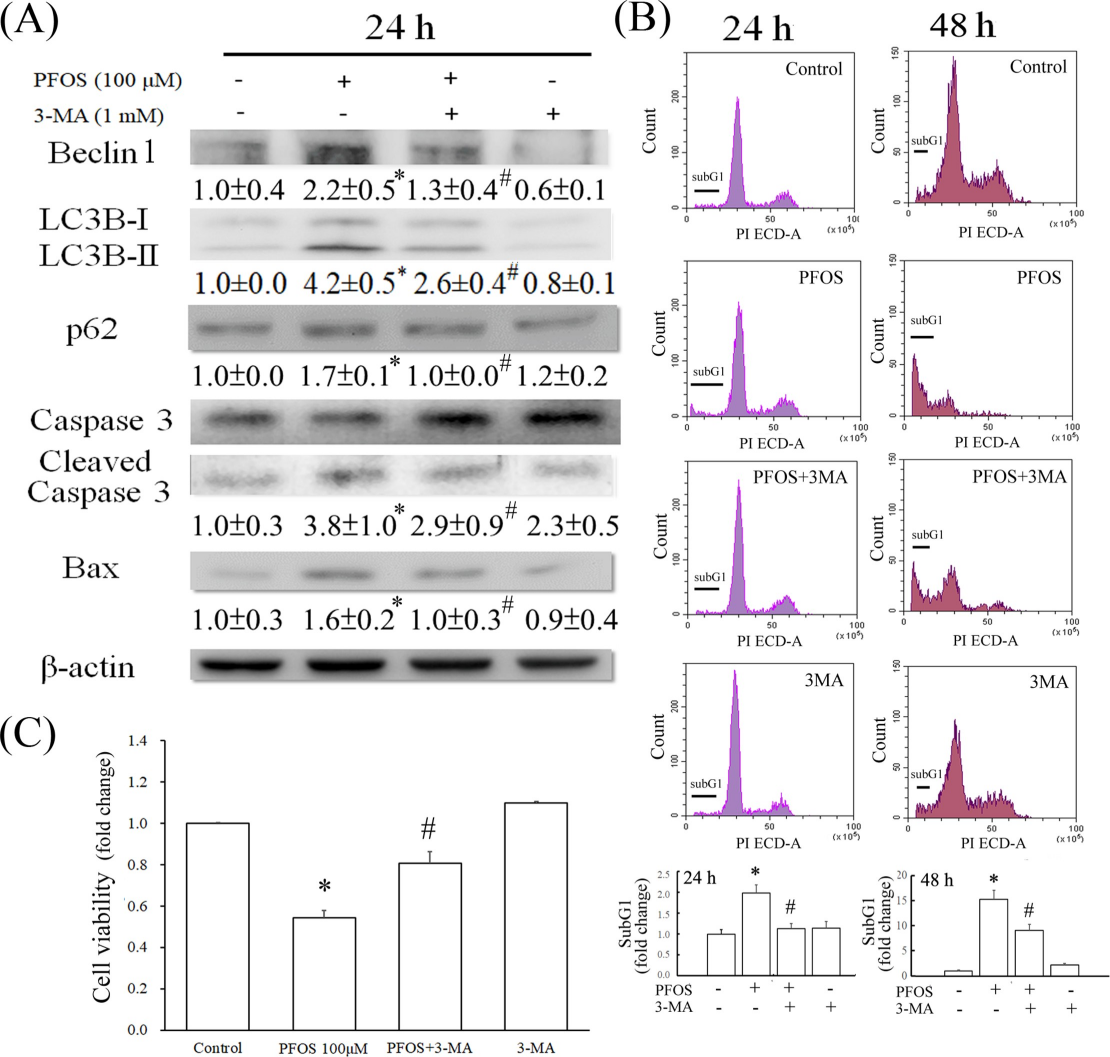
Autophagy-associated apoptosis in PFOS-treated RTCs. The effect of autophagy on PFOS-mediated RTC apoptosis was examined using 3MA (an early stage autophagy inhibitor). (A) Cells were pretreated with 1 mM 3MA for 2 h, followed by 100 μM PFOS for 24 h. Western blot analysis was used to examine the expression levels of the proteins involved in autophagy and apoptosis. (B) Percentage of the sub-G1 phase of the cell cycle according to flow cytometry analysis. Cells were incubated in 0.5% serum DMEM for 24 h to induce quiescence, followed by 100 μM PFOS treatment for 24 h or 48 h with or without 2 h of 1 mM 3MA pretreatment in 5% FBS DMEM medium. The sub-G1 phase and cell cycle were analyzed. The cell number is shown on the y-axis, and PI energy-coupled dye labeling (DNA content) is shown on the x-axis. Results of a representative experiment are shown. (C) Cell viability analysis using an MTT assay. The data are representative of the results of three independent experiments and are presented as the mean ± SEM (*P < 0.05 vs. control; ^#^*P* < 0.05 vs. PFOS treatment alone).

**Fig 3:**
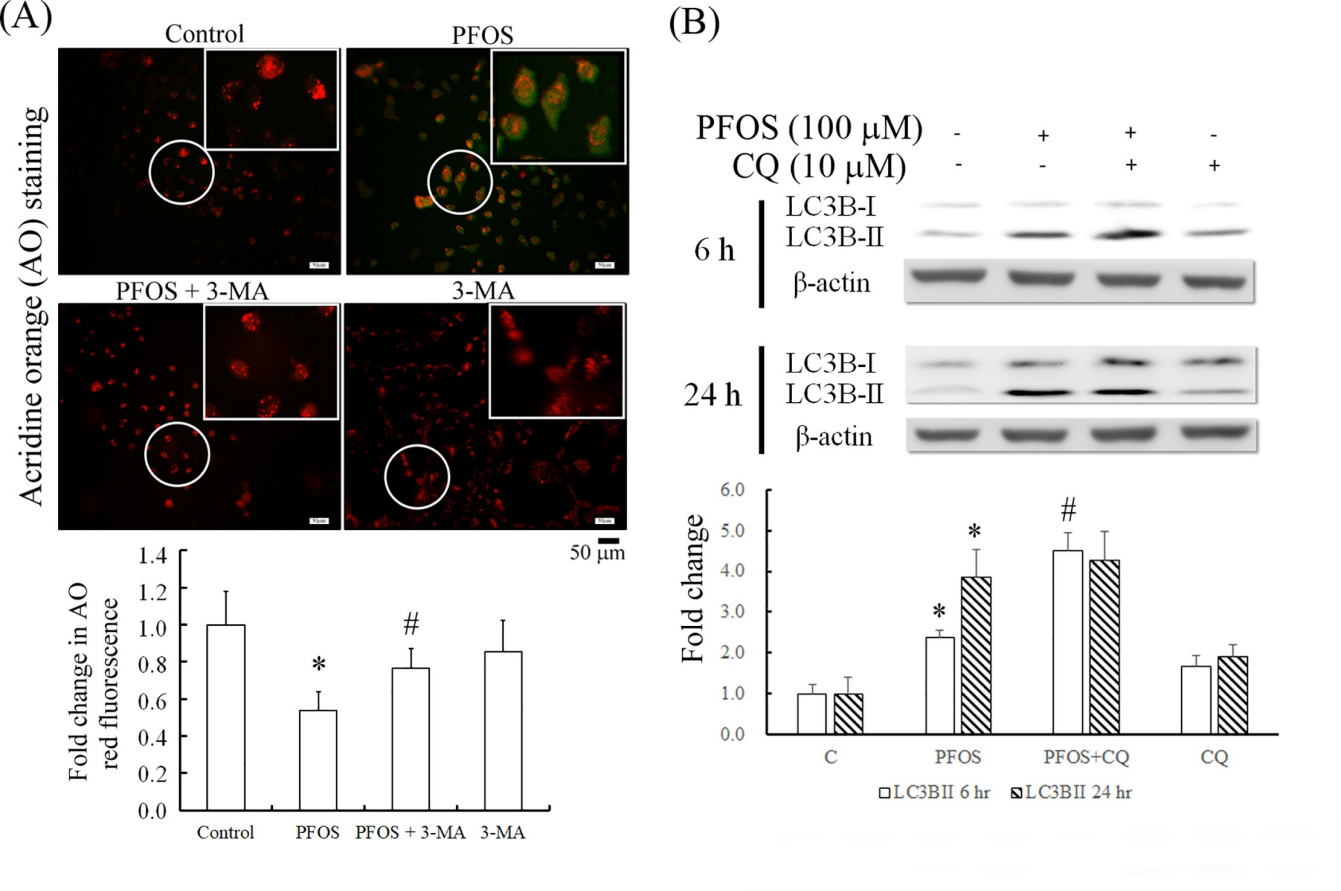
Time-dependent effect of PFOS on autophagy induction, followed by blocked autophagic flux. (A) The effect of PFOS on LMP was assessed using AO staining in RTCs with or without 3MA pretreatment. Cells grown on coverslips were pretreated with 3MA for 2 h, followed by 24-h PFOS treatment. The resulting cells were stained with AO dye for 30 min. The image displays representative images obtained using a fluorescence microscope. Magnification 100×, scale bar 50 μm. The cells in the white squares on the upper right sides of the panels are magnified images of those in white circles. (B) Whether autophagy induction or attenuated autophagic flux caused PFOS to increase LC3BII accumulation was investigated through CQ pretreatment. Cells were also subjected to 1-h pretreatment with CQ, an inhibitor of autolysosome formation, in PFOS-treated RTCs. Western blot was performed to examine the effect of CQ on LC3BII level after 6- and 24-h PFOS treatments. Bar charts show the band intensities of indicated proteins normalized using densitometry with β-actin. The data are representative of the results of three independent experiments and are presented as mean ± SEM (*P < 0.05 vs. control; ^#^*P* < 0.05 vs. PFOS treatment alone).

The causal relationship between autophagy and apoptosis was investigated using 3MA, an autophagy inhibitor. As illustrated in Fig 2A, pretreatment with 3MA reduced the protein levels of LC3BII, p62, and apoptotic biomarkers Bax and cleaved caspase 3 in PFOS-treated RTCs at 24 h. PFOS increased the subG1 phase of the cell cycle, as observed in the flow cytometry analysis (Fig 2B), and reduced cell viability, as observed in the MTT assay (Fig 2C), which could be rescued by 3MA pretreatment. Prolonged treatment with PFOS increased LMP, as indicated by increased green staining with AO dye instead of general red staining (pH of approximately 4.5), but this could be attenuated by pretreatment with 3MA (Fig 3A). These results suggest that PFOS-induced lysosome impairment depended on autophagy induction. Accordingly, PFOS induced autophagy at 6 h (Fig 1) and reduced autophagic flux by impairing lysosome integrity after 24 h (Fig 3A). CQ, an inhibitor of autolysosome formation, was used to examine whether LC3BII accumulation in response to PFOS administration occurred due to the activation of autophagy or the attenuation of the autophagic flux. Fig 3B demonstrates that additional treatment with CQ further increased LC3BII accumulation compared with only PFOS treatment after 6 h of autophagy induction, suggesting that PFOS can initiate autophagy activation. However, compared with LC3BII levels after a 24-h treatment with PFOS, an additional CQ pretreatment exhibited no apparent alteration in LC3BII accumulation.

### Roles of ROS and extracellular-signal-regulated kinase 1/2 activation in PFOS-mediated autophagy and apoptosis

**Fig 4:**
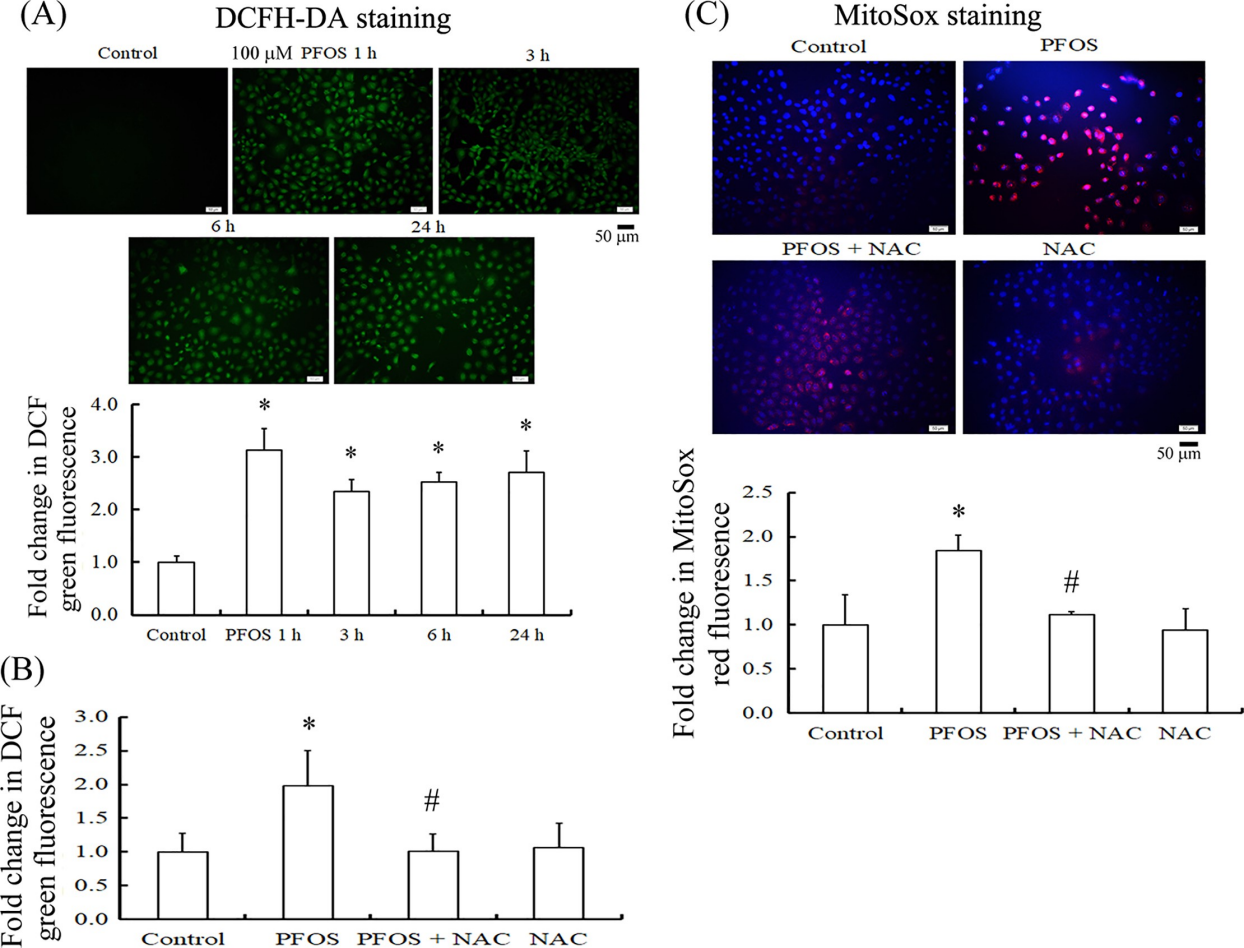
Alleviation of increased generation of cytosolic and mitochondrial ROS by NAC in PFOS-treated RTCs. ROS induced by PFOS were detected in the cytosol at 1–24 h and in mitochondria after 24 h of treatment. (A) Cells were treated with 100 μM at 1, 3, 6, and 24 h; cytosolic ROS production was assayed through DCFH-DA staining. (B) The effect of PFOS on cytosolic ROS production at 24 h was assessed for the intervention using 2 h of 100 μM NAC pretreatment. (C) The effect of PFOS on mitochondrial ROS production was investigated using MitoSox staining followed by 5 min of DAPI staining for nuclei; the effect was evaluated through the addition of NAC pretreatment to PFOS-treated RTCs for 24 h. Data are representative of the results of three independent experiments. The quantified data are shown in bar graphs and are presented as mean ± SEM. (*P < 0.05 vs. control; ^#^*P* < 0.05 vs. PFOS treatment alone).

ROS are involved in autophagic signaling [18], and PFOS induces ROS production [19]. PFOS significantly increased ROS production after 1, 3, 6, and 24 h of treatment, as observed using the DCFH-DA staining procedure (Fig 4A). The oxidative stress effect of 24-h PFOS treatment in RTCs was significantly attenuated by NAC treatment (Fig 4B). Furthermore, after 24-h PFOS treatment, MitoSox staining revealed increased mitochondrial ROS, which could be prevented by additional pretreatment with NAC, the quantitation for which is displayed in Fig 4C.

**Fig 5:**
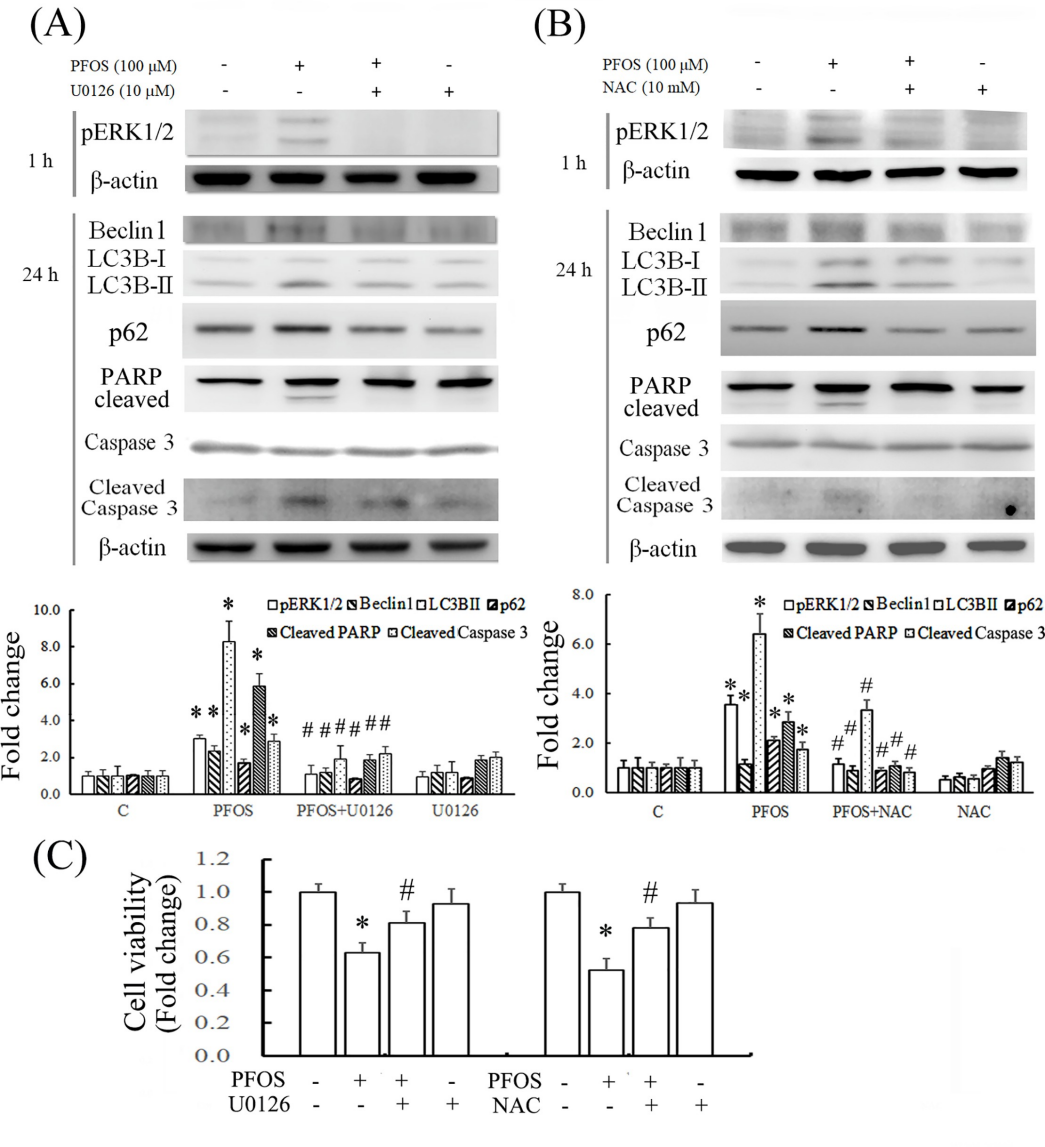
Inhibition of autophagy-associated apoptosis by U0126 and NAC through the reduction of ERK1/2 phosphorylation in PFOS-treated RTCs. Cells were pretreated with 10 μM U0126 (A) for 1 h and 10 mM NAC (B) for 2 h, followed by PFOS treatment for 1 and 24 h and examination using Western blot analysis for indicated molecules. The protein bands of interest were quantified and plotted in the bar graph. The bar charts show the band intensities of indicated proteins using densitometry and normalized with β-actin. The data are representative of the results of three independent experiments, and data are presented as the mean ± SEM (*P < 0.05 vs. control; ^#^*P* < 0.05 vs. PFOS treatment alone). (C) Cell viability was measured using an MTT assay. The data are representative of the results of three independent experiments, and the data are presented as the mean ± SEM (*P < 0.05 vs. control; ^#^*P* < 0.05 vs. PFOS treatment alone).

We further explored the downstream targets of ROS induction in RTCs treated with PFOS; various mitogen-activated protein kinase (MAPK) pathways could be associated with it. The signaling pathway of extracellular-signal-regulated kinase (ERK) was sought for its response to the PFOS challenge. PFOS treatment for 1 h significantly activated ERK1/2 through phosphorylation (Fig 5A). In addition, ERK1/2 inactivation by U0126 attenuated autophagy induction by PFOS, as indicated by the attenuated levels of Beclin 1, LC3BII, and p62 proteins after 24 h of treatment, followed by a reduction in cleaved PARP and caspase 3 levels (Fig 5A). In addition, NAC, an ROS scavenger, inhibited PFOS-mediated autophagy induction through ERK1/2 inactivation by reducing the level of ERK1/2 phosphorylation and the protein levels of Beclin 1, LC3BII, and p62, which subsequently reduced cleaved PARP and caspase 3 levels (Fig 5B). The effect of these inhibitors in combination with PFOS on cell viability was examined using the MTT assay, which demonstrated that U0126 and NAC significantly rescued cells from PFOS-mediated decreases in cell viability (Fig 5C).

## Discussion

Perfluorinated compounds (PFCs), particularly PFOS, are used in everyday life and are ubiquitously distributed in the environment, which has caused concerns lately due to their correlation with the incidence of many diseases [20]. In humans, the PFOS elimination half-life in serum is approximately 8.7 years; this was recently estimated from retired fluorochemical workers [21]. In the serum of production workers, PFOS concentrations as high as 12.83 ppm (approximately 23.8 μM) have been reported [22], with the highest reported level of 26 μM [23, 24]. In the present study, we used 100 μM PFOS, which is higher than the average exposure; however, we chose this concentration (four times that in production workers) because of the short duration of PFOS exposure (1, 6, and 24 h) to RTCs. In other studies, PFOS concentrations of as high as 300 μM have been used for apoptosis and autophagy activation in hepatocytes and in adipocyte differentiation [4, 7, 25]. Additionally, a positive correlation exists between increased serum PFOS levels and renal function impairment (e.g., creatinine, estimated glomerular filtration rate (eGFR), and uric acid) and chronic kidney disease [26, 27]. Therefore, the potential toxicity of PFOS to humans must be intensively evaluated to avoid long-term accumulation.

In a previous study, we reported that PFOS could induce RTC apoptosis by increasing oxidative stress [2]. In the present study, we extended our findings and elucidated the relationship between autophagy and apoptosis in PFOS-treated cells. We demonstrated that PFOS induced autophagy after as early as 6 h, leading to RTC death, but this could be attenuated by pretreatment with 3MA, an autophagy inhibitor. PFOS has been reported to cause apoptosis in human hepatoma HepG2 cells [7] and in primary rat cerebellar granule cells through ROS-dependent protein kinase C signaling [28]. Our data indicated that PFOS induced cytosolic and mitochondrial ROS production (Fig 4), both of which could be rescued by NAC, which suppressed autophagy induction and apoptosis (Fig 5B). Nevertheless, crosstalk occurs between apoptosis and autophagy: autophagy generally mitigates apoptosis induction, whereas apoptosis-related caspase activation blocks autophagy. Unequivocally, autophagy or autophagy-associated protein facilitates apoptosis induction, with autophagy generally preceding apoptosis. Therefore, controversial results may be observed depending on the experimental settings.

We previously concluded that oxidative stress mediates PFOS-induced apoptosis in RTCs [2]. Our current study demonstrated that autophagosome formation was activated in the early stage of PFOS treatment (6 h), and autophagosome degradation was impaired in the late stage (24 h). In addition, the accumulation of p62 protein (an autophagy substrate) was increased after 24 h of PFOS treatment due to the impairment of lysosomal integrity (Fig 2A), visualized as decreased red fluorescence with AO staining (Fig 3A) and indicating increased LMP levels. p62 is a scaffolding protein that interacts with various partners; in particular, its binding with LC3BI and ubiquitin is associated with the protein degradation involved in autophagy [10]. It also activates the death receptor pathway by promoting the aggregation of cullin-3-modified caspase-8, resulting in apoptosis [29]. This might be another mechanism by which PFOS affects RTCs. Consistent with our findings, a study revealed that PFOS attenuates autophagic flux by increasing LMP and autophagosome accumulation, leading to the death of primary neurons and brain astrocyte cells in rats [30]. Another study also demonstrated similar effects in MCF-7 cells by using nanoparticles of iron oxide [31]. Notably, a PFOS-induced increase in LMP could be mitigated through additional 3MA treatment, indicating the dependence on LMP for autophagy induction (Fig 3A). In addition, the blocking of autolysosome formation by CQ at an early stage of autophagy activation (6-h PFOS treatment) induced LC3B II accumulation in RTCs (Fig 3B), suggesting that PFOS initiated autophagosome formation after autolysosome formation was impaired by CQ.

PFOS-mediated RTC apoptosis resulted from autophagy activation through ROS-related ERK1/2 activation (Fig 5). The MAPK pathway is involved in endoplasmic reticulum stress, cell proliferation, cell cycle arrest, and autophagy [32]. In addition, PFOS can induce ERK activation and ER stress [33]. ERK1/2 activation can increase Beclin 1 expression and, subsequently, induce autophagy [34]. However, autophagy can prompt either cell death or cell survival, depending on cell type and circumstances. For example, triptolide was reported to induce autophagy and cell death in the breast cancer cell line MCF-7 through increased ERK1/2 phosphorylation [34], and cisplatin caused autophagy and cell death in mouse renal tubular epithelial cells, EpiCM-a cells, through ERK1/2 activation [35]. By contrast, aristolochic acid I attenuated cell death through pERK1/2-mediated autophagy activation in rat RTCs (NRK-52E) [36]. Therefore, even in the same types of cells (i.e., RTCs) and with a similar signaling pathway, autophagy can either cause cell death or cell survival.

**Fig 6:**
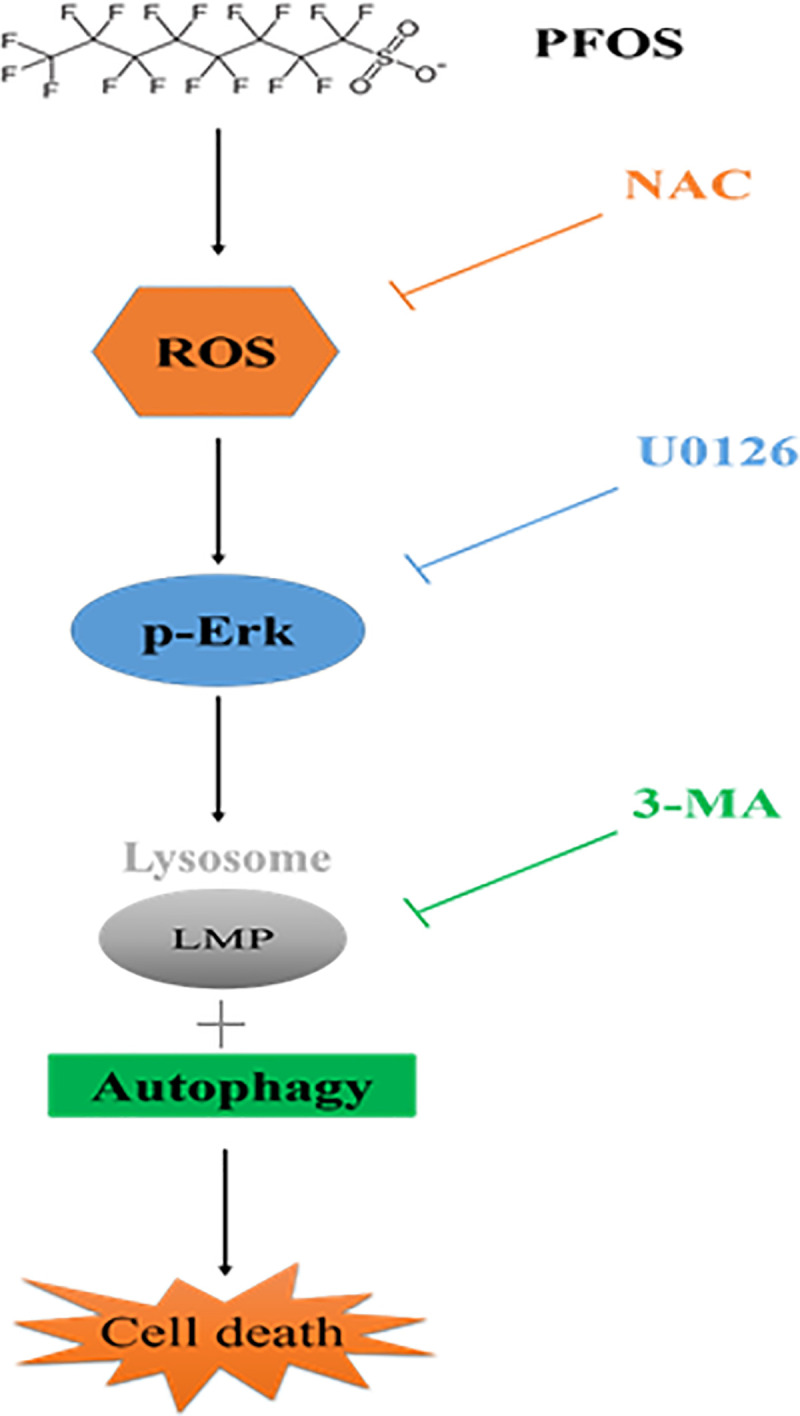
The mechanism underlying the effect of autophagy activation on RTC apoptosis caused by PFOS. The effect of autophagy induction on apoptosis through ROS and pERK1/2 signaling in PFOS-treated RTCs. PFOS can induce autophagy activation through a short treatment, followed by a decreased autophagic flux due to increased LMP caused by prolonged PFOS treatment of RTCs. These effects lead to apoptosis in PFOS-treated RTCs. The sequential addition of NAC, U0126, and 3MA can eliminate the effect of PFOS in a stepwise manner on autophagy-associated apoptosis through ROS and ERK1/2 inactivation.

We previously demonstrated that PFOS can damage RTCs and murine kidneys through oxidative stress and inflammation-related mechanisms [2]. The current study further revealed that the apoptotic event is associated with autophagy activation by PFOS. Notably, the autophagy inhibitors 3-MA, NAC, and U0126 (an inhibitor of the MAPK kinases) could protect cells from PFOS-mediated increased LMP and decreased cell viability (as summarized in Fig 6). Accordingly, we revealed that PFOS potentially caused RTC apoptosis through autophagy activation primarily through ROS-mediated pERK1/2 activation. Our findings thus clarified the mechanism of PFOS-mediated renal toxicity and may inform further research on relevant clinical applications.

## Supporting information

S1 Raw images (PDF)
