# Supplementary material for: Perfluorooctane sulfonate induces autophagy-associated apoptosis through oxidative stress and the activation of extracellular signal–regulated kinases in renal tubular cells
Source: PLoS One. 2021 Jan 20;16(1):e0245442. doi: 10.1371/journal.pone.0245442 (PMC7817024; doi:10.1371/journal.pone.0245442)
Supplement: S1 Raw images — (PDF) [file pone.0245442.s001.pdf]

Fig 1A

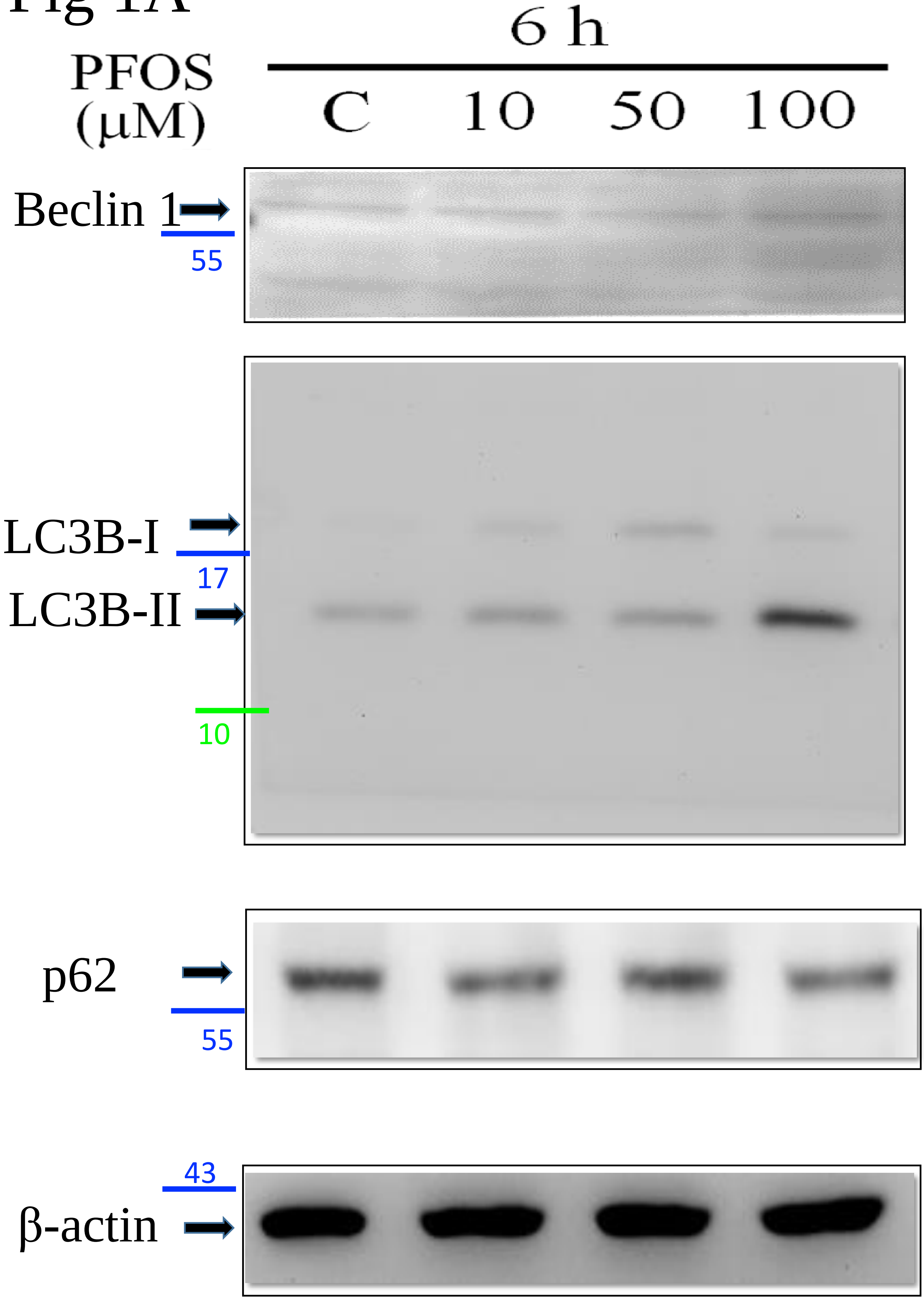
6 h
PFOS (μM)
C
10
50
100
Beclin 1
55
LC3B-I
17
LC3B-II
10
p62
55
43
β-actin

Fig 1B_1

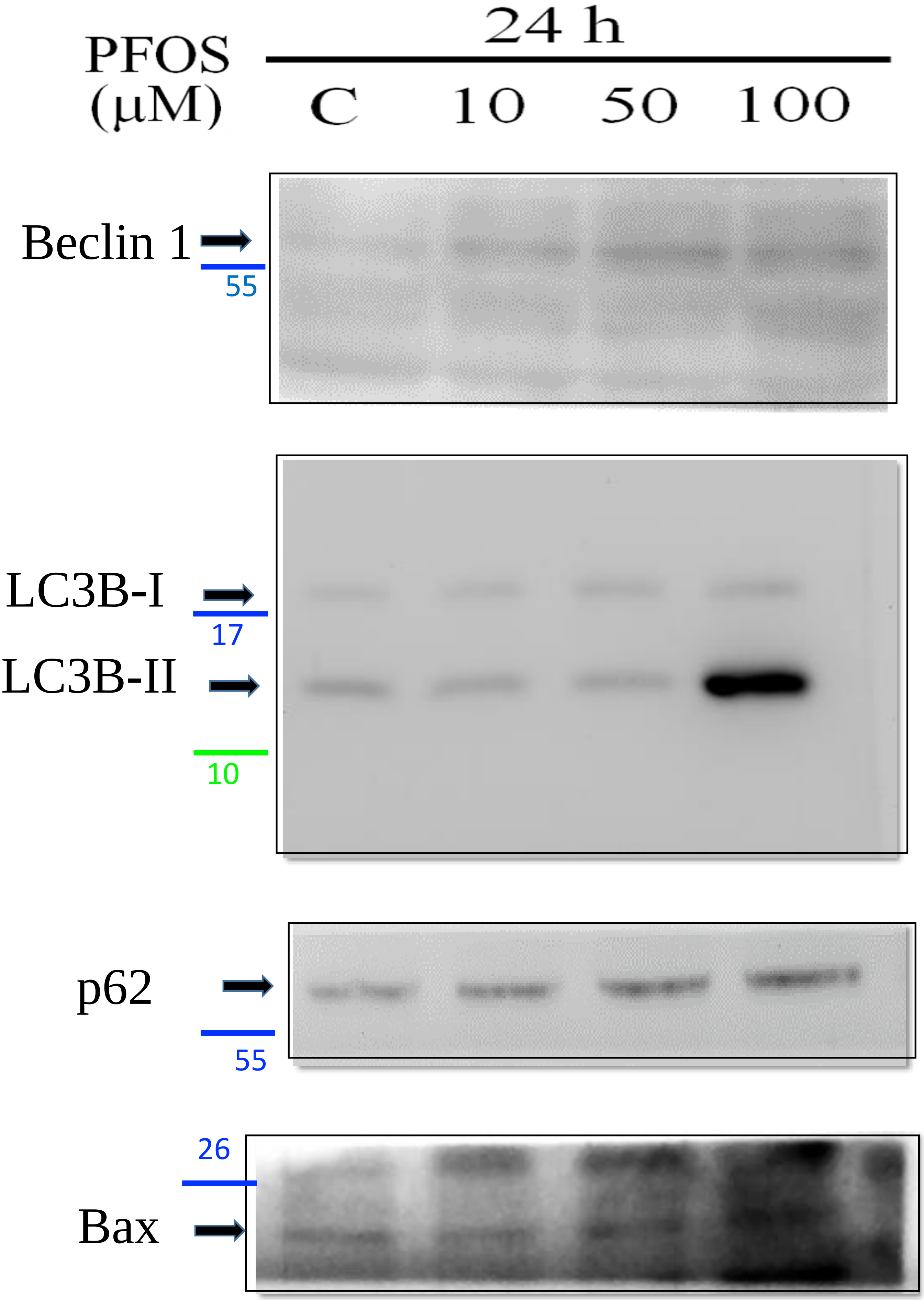

24 h
PFOS
(μM)
C
10
50
100
Beclin 1
55
LC3B-I
17
LC3B-II
10
p62
55
26
Bax

Fig 1B_2

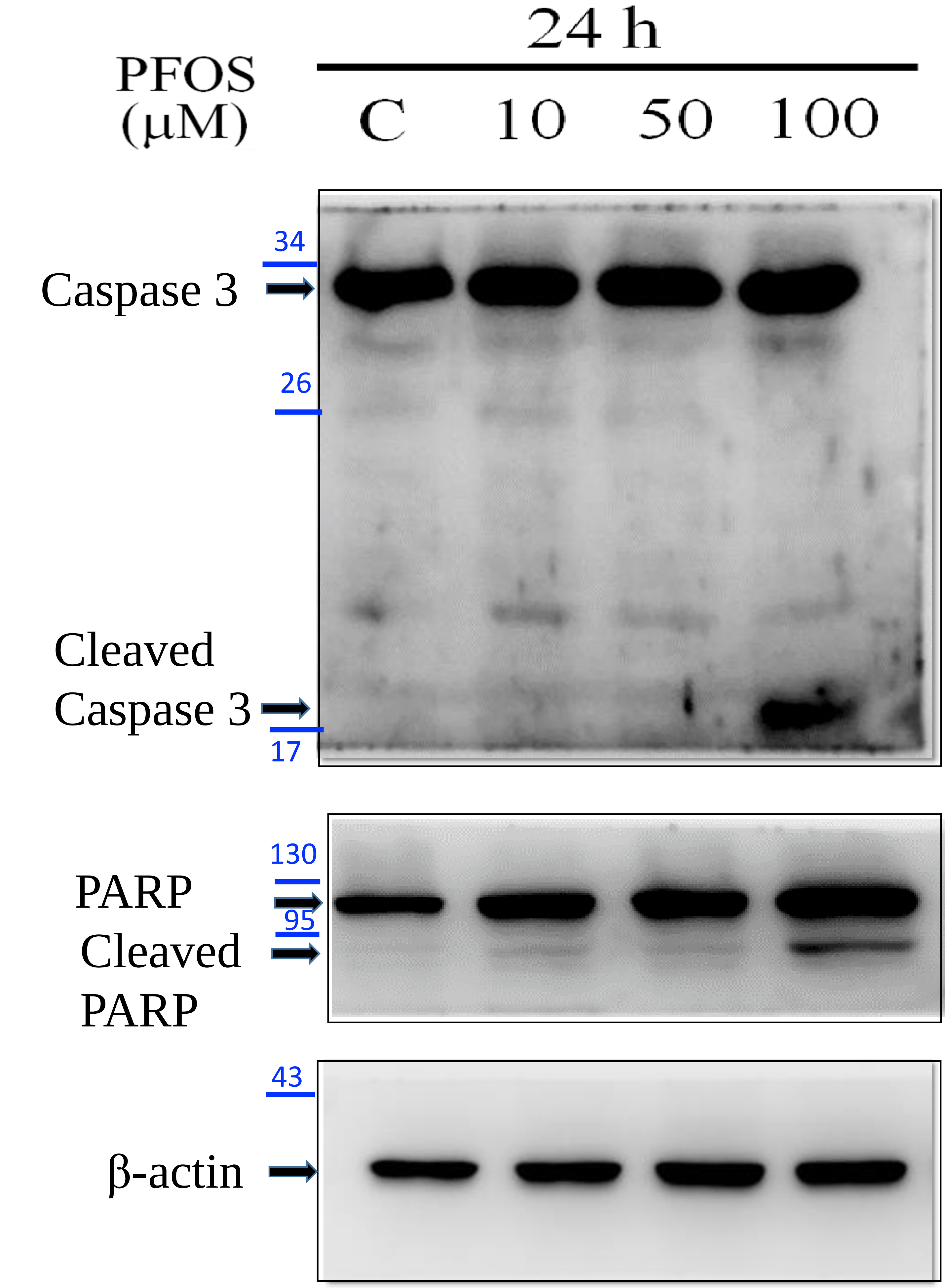

24 h
PFOS (μM)
C
10
50
100
34
Caspase 3
26
Cleaved Caspase 3
17
130
PARP
95
Cleaved PARP
43
β-actin

Fig 1C

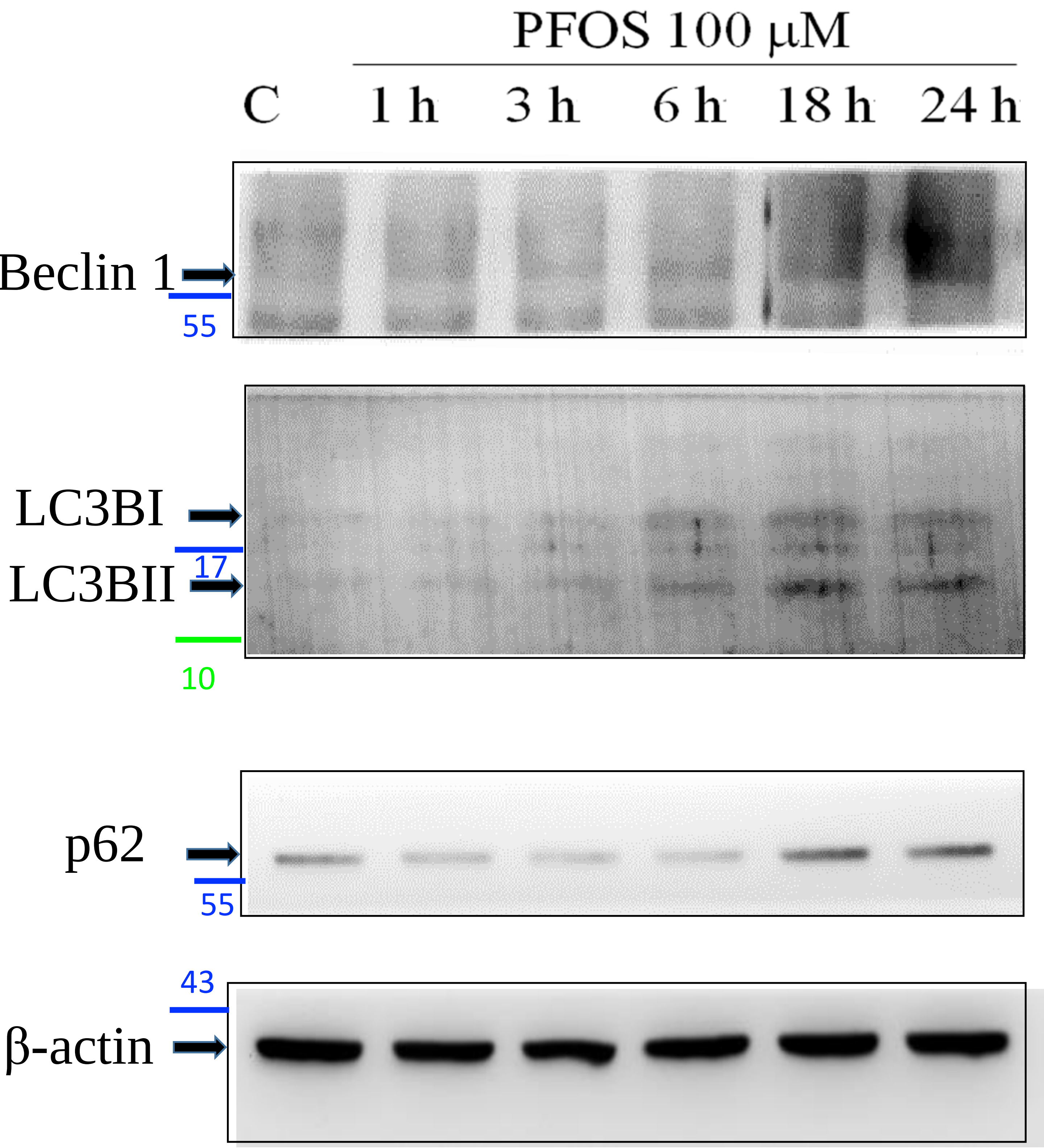
PFOS 100 μM
C
1 h
3 h
6 h
18 h
24 h
Beclin 1
55
LC3BI
17
LC3BII
10
p62
55
43
β-actin

Fig 2A_1

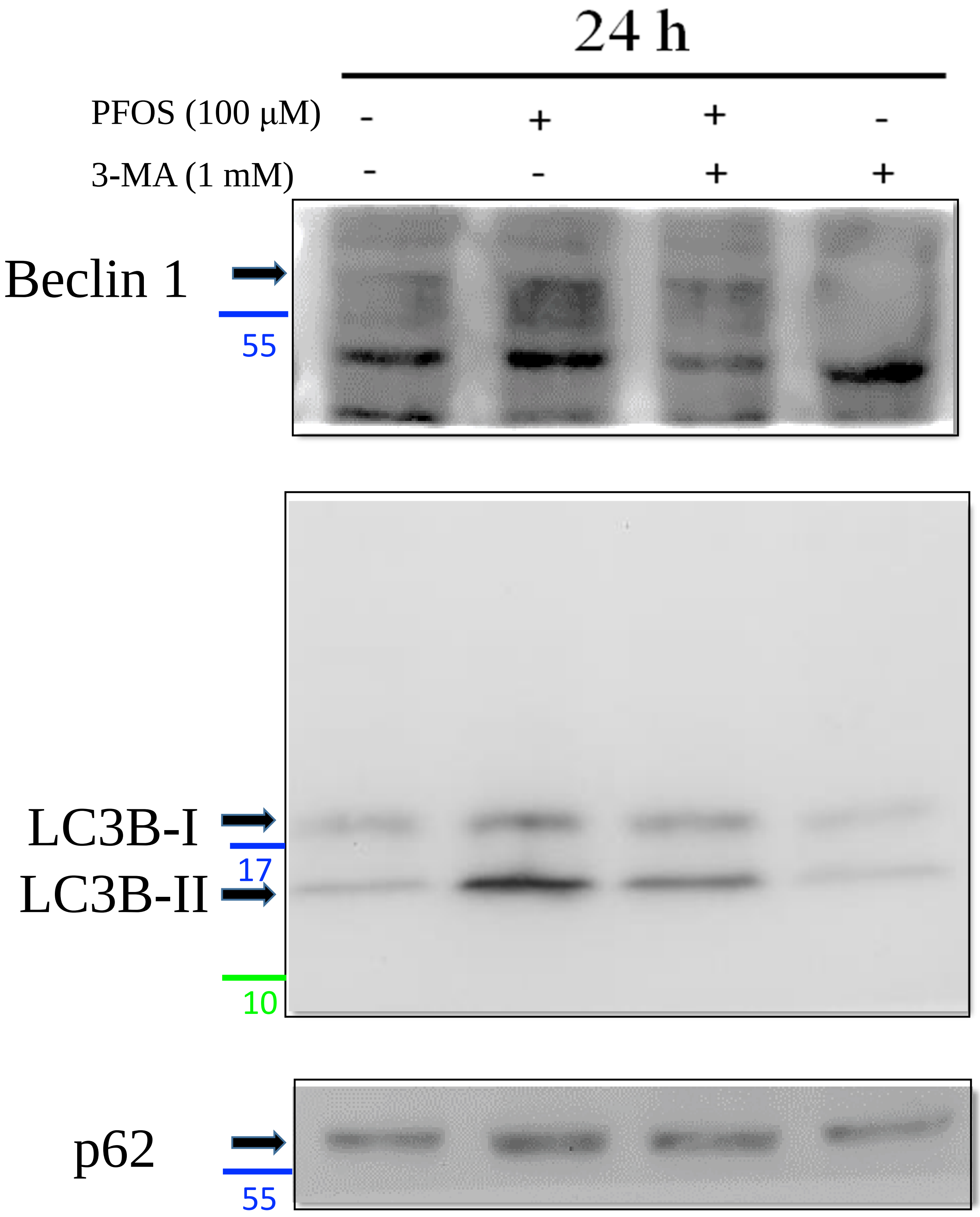

Fig 2A_2

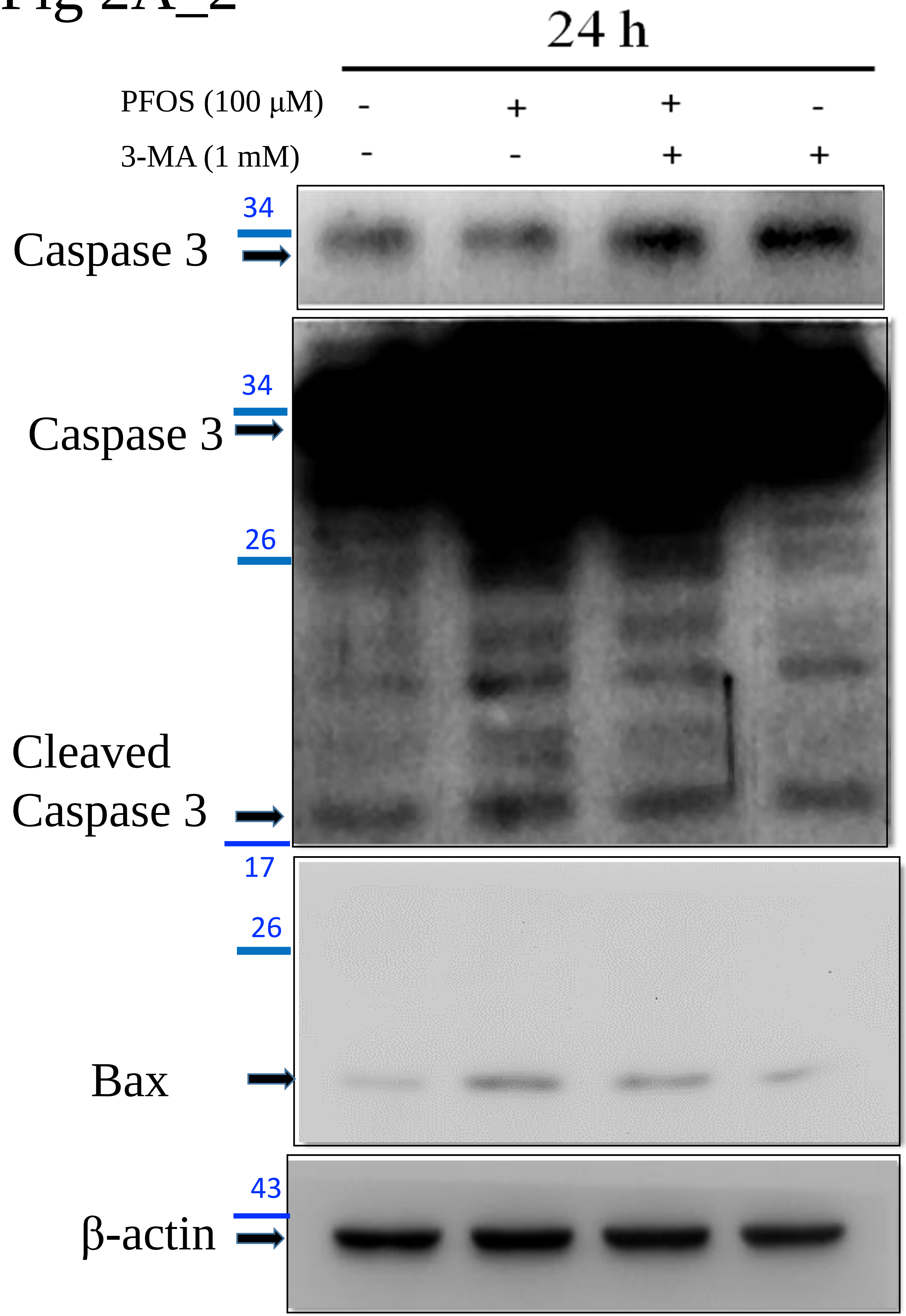
24 h
PFOS (100 μM) - + + -
3-MA (1 mM) - - + +
34
Caspase 3
34
Caspase 3
26
Cleaved
Caspase 3
17
26
Bax
43
β-actin

Fig 3B

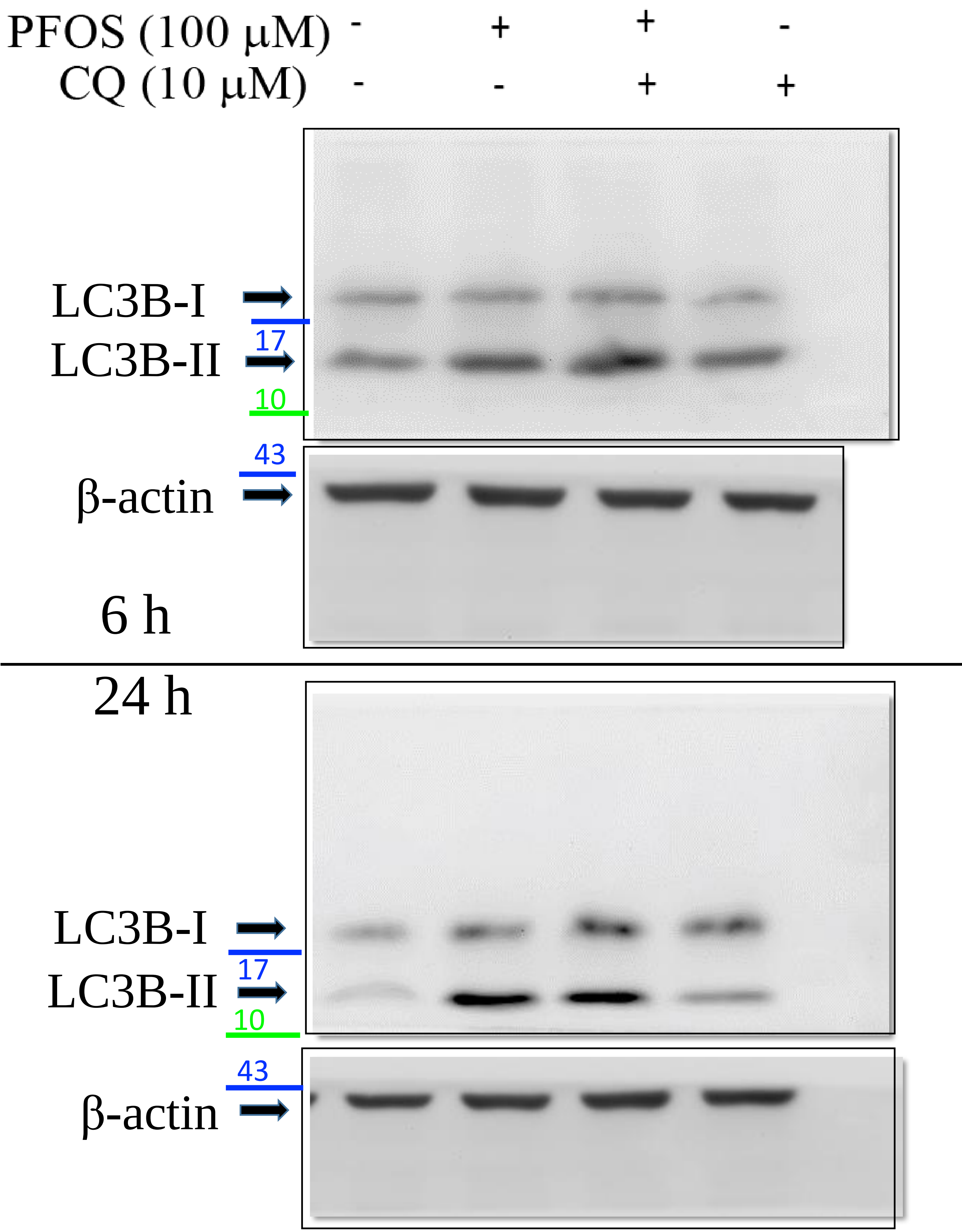
PFOS (100 μM) - + + -
CQ (10 μM) - - + +
LC3B-I
17
LC3B-II
10
43
β-actin
6 h
24 h
LC3B-I
17
LC3B-II
10
43
β-actin

Fig 5A_1

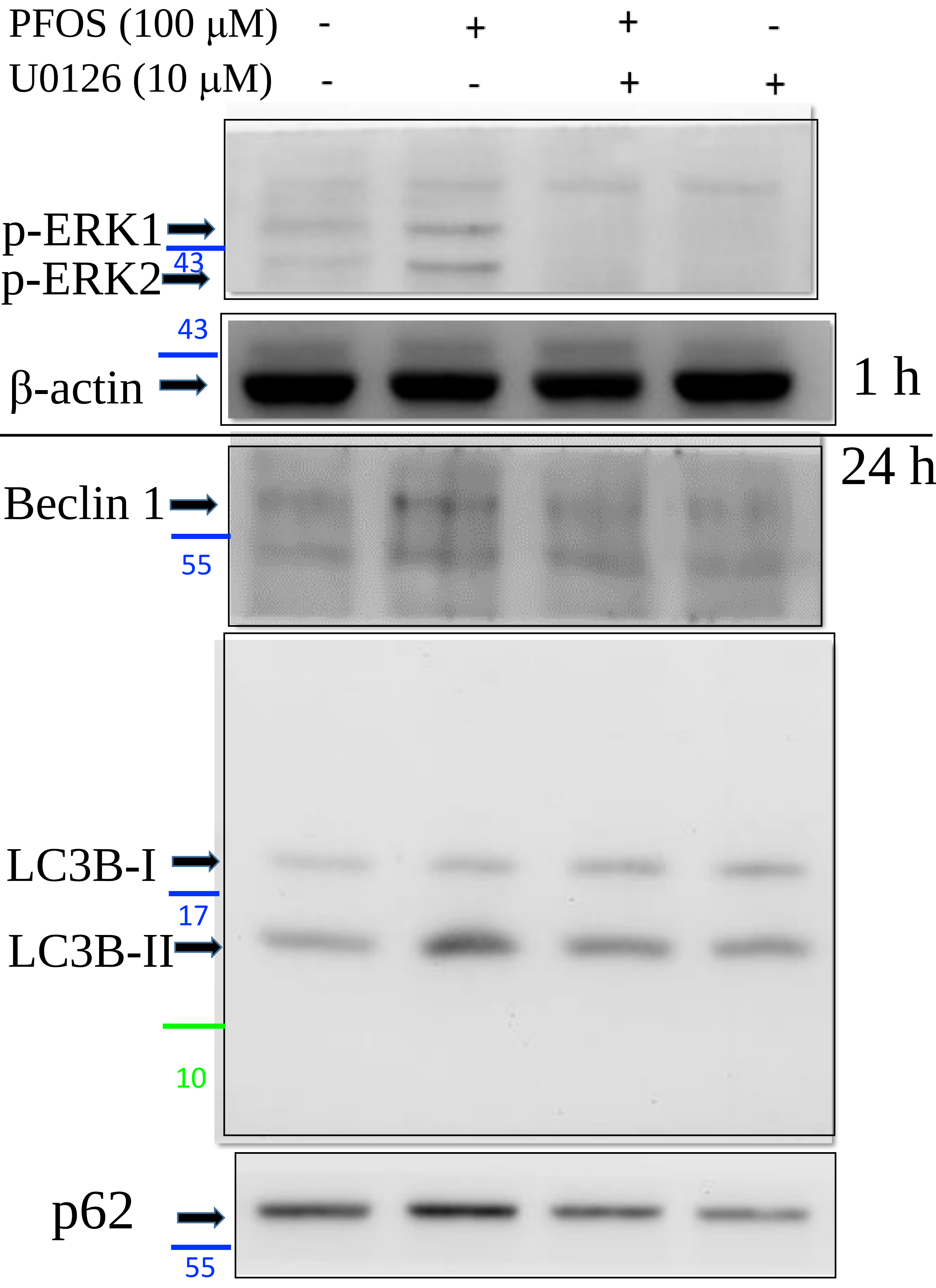

PFOS (100 μM) - + + -
U0126 (10 μM) - - + +
p-ERK1
43
p-ERK2
43
β-actin
1 h
24 h
Beclin 1
55
LC3B-I
17
LC3B-II
10
p62
55

Fig 5A_2

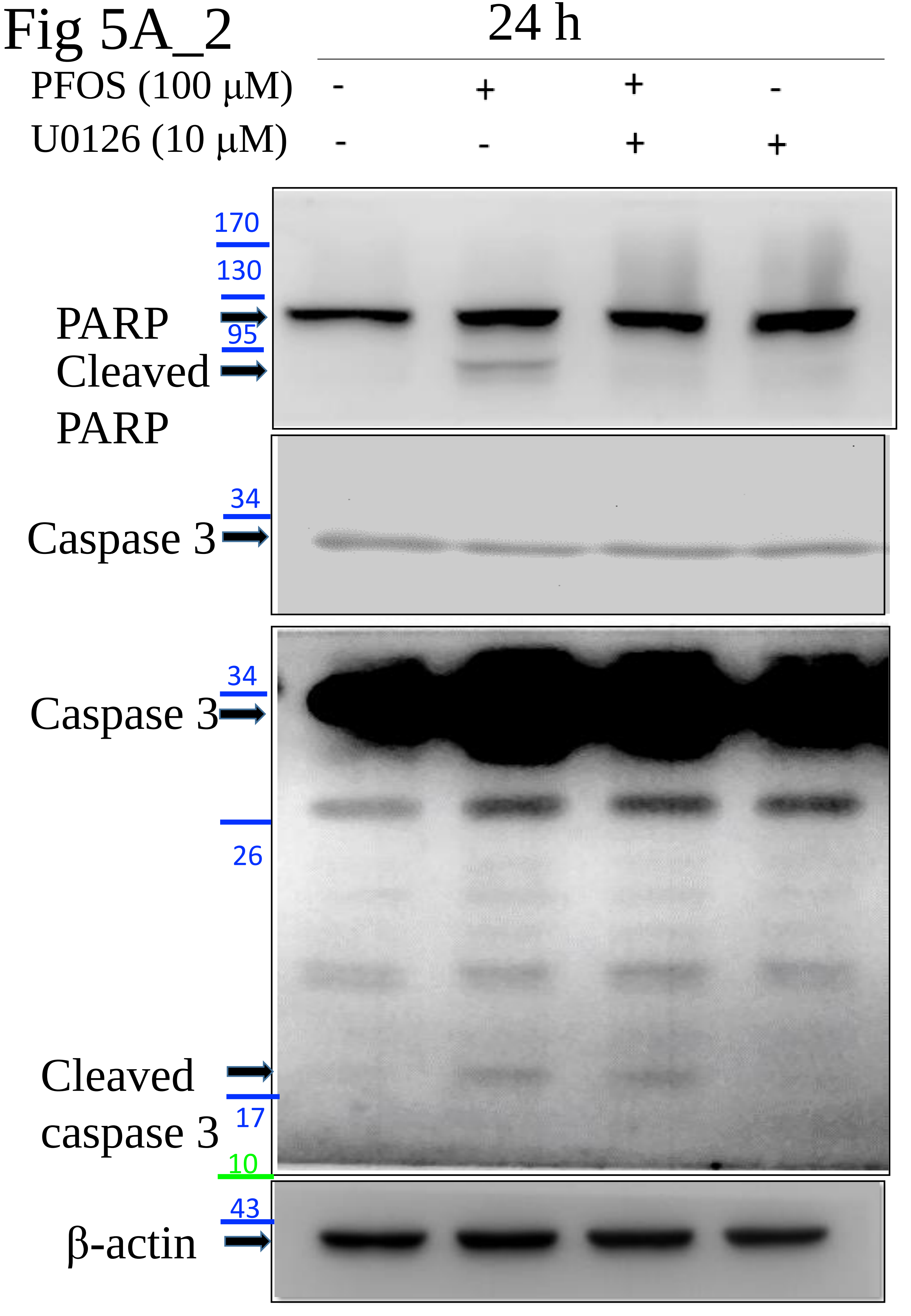

24 h
PFOS (100 μM) - + + -
U0126 (10 μM) - - + +
170
130
PARP
95
Cleaved PARP
34
Caspase 3
34
Caspase 3
26
Cleaved caspase 3
17
10
43
β-actin

Fig 5B_1

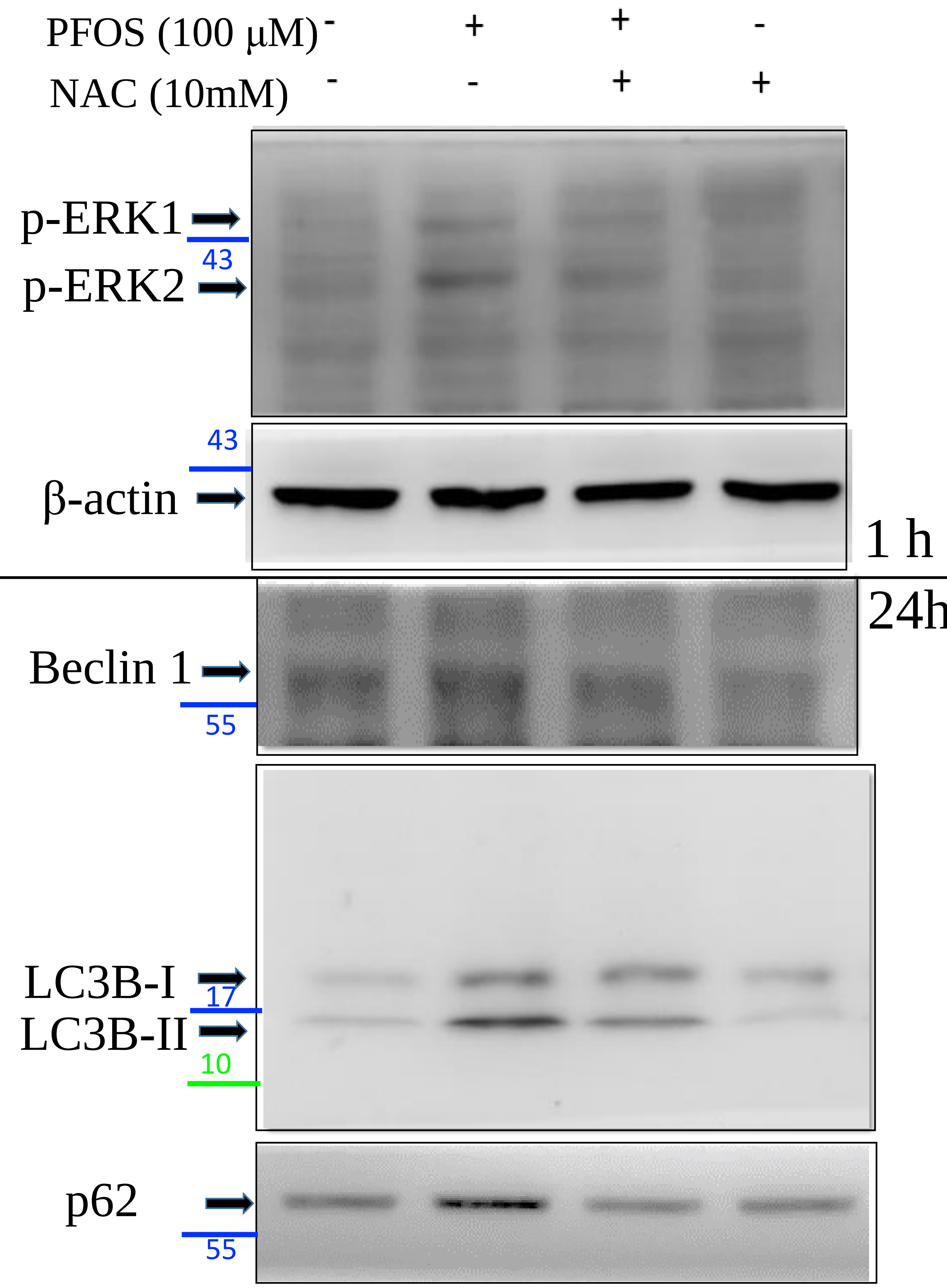
PFOS (100 μM) - + + -
NAC (10mM) - - + +
p-ERK1
43
p-ERK2
43
β-actin
1 h
24h
Beclin 1
55
LC3B-I
17
LC3B-II
10
p62
55

Fig 5B_2

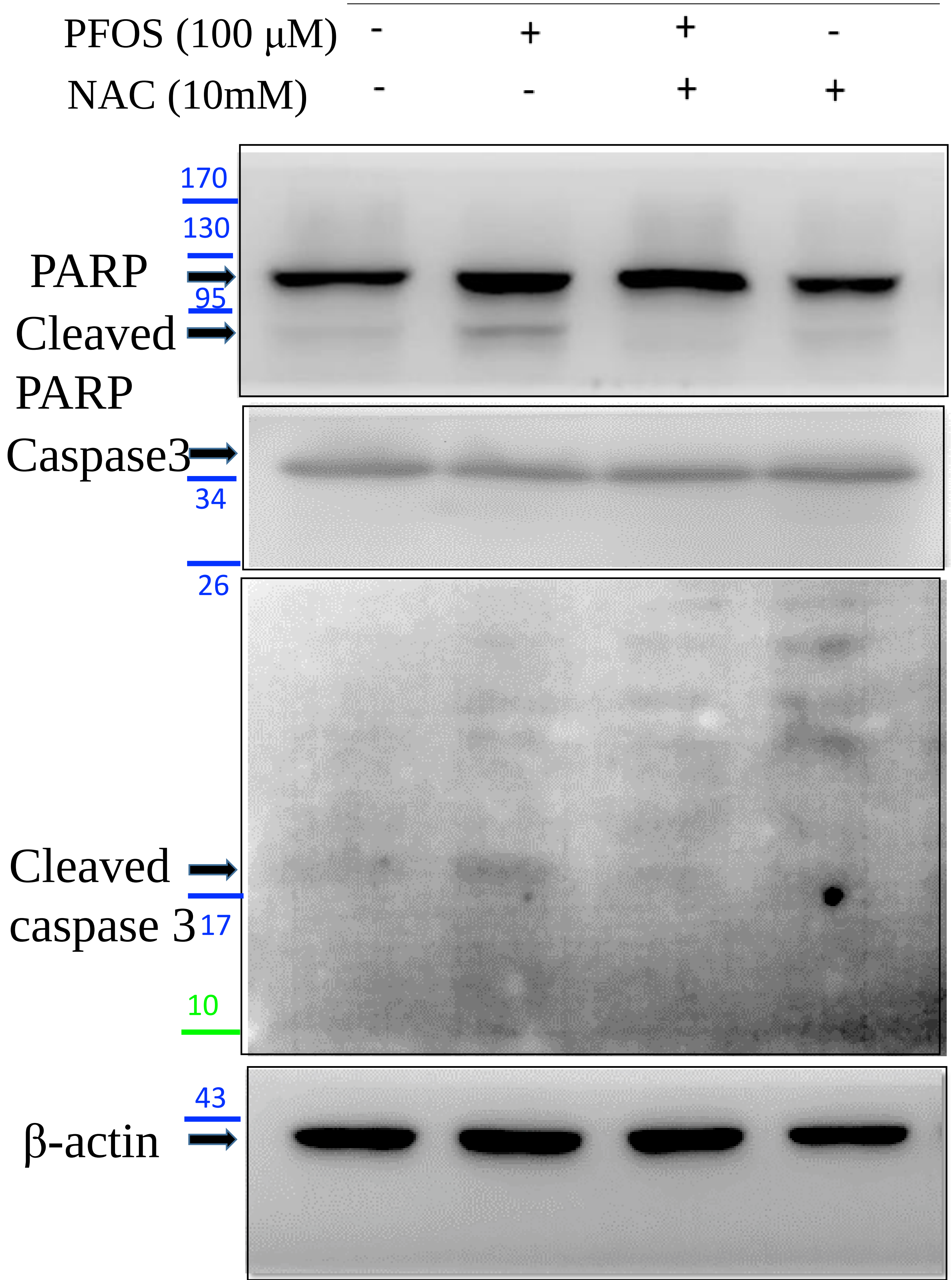

24 h
PFOS (100 μM) - + + -
NAC (10mM) - - + +
170
130
PARP
95
Cleaved PARP
Caspase3
34
26
Cleaved caspase 3
17
10
43
β-actin
